# Performance And Agreement Of Risk Stratification Instruments For Postoperative Delirium In Persons Aged 50 Years Or Older

**DOI:** 10.1371/journal.pone.0113946

**Published:** 2014-12-02

**Authors:** Carolien J. Jansen, Anthony R. Absalom, Geertruida H. de Bock, Barbara L. van Leeuwen, Gerbrand J. Izaks

**Affiliations:** 1 University of Groningen, University Medical Center Groningen, University Center for Geriatric Medicine, Groningen, the Netherlands; 2 University of Groningen, University Medical Center Groningen, Department of Anesthesiology, Groningen, the Netherlands; 3 University of Groningen, University Medical Center Groningen, Department of Epidemiology, Groningen, the Netherlands; 4 University of Groningen, University Medical Center Groningen, Department of Surgery, Groningen, the Netherlands; D'or Institute of Research and Education, Brazil

## Abstract

Several risk stratification instruments for postoperative delirium in older people have been developed because early interventions may prevent delirium. We investigated the performance and agreement of nine commonly used risk stratification instruments in an independent validation cohort of consecutive elective and emergency surgical patients aged ≥50 years with ≥1 risk factor for postoperative delirium. Data was collected prospectively. Delirium was diagnosed according to DSM-IV-TR criteria. The observed incidence of postoperative delirium was calculated per risk score per risk stratification instrument. In addition, the risk stratification instruments were compared in terms of area under the receiver operating characteristic (ROC) curve (AUC), and positive and negative predictive value. Finally, the positive agreement between the risk stratification instruments was calculated. When data required for an exact implementation of the original risk stratification instruments was not available, we used alternative data that was comparable. The study population included 292 patients: 60% men; mean age (SD), 66 (8) years; 90% elective surgery. The incidence of postoperative delirium was 9%. The maximum observed incidence per risk score was 50% (95%CI, 15–85%); for eight risk stratification instruments, the maximum observed incidence per risk score was ≤25%. The AUC (95%CI) for the risk stratification instruments varied between 0.50 (0.36–0.64) and 0.66 (0.48–0.83). No AUC was statistically significant from 0.50 (*p*≥0.11). Positive predictive values of the risk stratification instruments varied between 0–25%, negative predictive values between 89–95%. Positive agreement varied between 0–66%. No risk stratification instrument showed clearly superior performance. In conclusion, in this independent validation cohort, the performance and agreement of commonly used risk stratification instruments for postoperative delirium was poor. Although some caution is needed because the risk stratification instruments were not implemented exactly as described in the original studies, we think that their usefulness in clinical practice can be questioned.

## Introduction

As a result of changing population demographics, an increasing number of older patients are undergoing surgery. It is estimated that in 2020, the number of surgical procedures performed in persons aged 65 years or older in the United States will be 14% to 47% higher (dependent on specialty) than in 2001 [1]. Importantly, more than 40% of the patients in this age group experience a major postoperative complication [2], of which postoperative delirium is among the most common [3]. Postoperative delirium is associated with worse outcomes in older patients but can be prevented by tailored interventions that address a number of modifiable risk factors [4]. Therefore, current guidelines recommend routine preoperative assessment of delirium risk in this age group [3].

For adequate assessment of postoperative delirium risk, reliable risk stratification instruments are essential. Ideally, a risk stratification instrument correctly identifies older surgical patients who are at increased risk of postoperative delirium and are likely to benefit from preoperative and postoperative interventions to prevent delirium [4]. *S*everal risk stratification instruments for delirium have been developed since the early 1990s [5]–[15]. Most of them are based on well-known risk factors for delirium, such as high age, cognitive impairment and alcohol abuse, and were found to have a very good to excellent performance. For some risk stratification instruments, the positive predictive value for incident delirium was 83 percent or higher [8],[11],[13]. Nevertheless, the generalizibility of many of these risk stratification instruments can be questioned because their performance has only been investigated in highly specific patient populations such as, for example, patients undergoing cardiac surgery [13], or patients with elective hip or knee arthroplasty [9], or hip fracture [10]. Furthermore, the validity of several risk stratification instruments has been tested in only one or two independent validation samples since their development [9]–[12]. Therefore, the performance and relevance of current risk stratification instruments for delirium is still unclear.

The aim of the study was to investigate in an independent validation sample, the performance of commonly used risk stratification instruments for postoperative delirium in older patients. The study sample included a total of 292 persons aged 50 years or older who underwent elective or emergency surgery.

## Methods

### Study Population

The study was performed at the University Medical Center Groningen, a 1,300 bed university hospital in the northern Netherlands. The study population included all consecutive elective and emergency surgical patients aged 50 years or older who were admitted between 1 October 2011 and 1 June 2012 and met at least one of the following inclusion criteria: memory problems; dependency in activities of daily living (ADL) during the last 24 hours; history of confusion during previous illness or hospitalization; alcohol abuse; thoracic or abdominal surgery; age ≥70 years (for emergency admission patients); planned ICU admission (for elective admission patients). The first three criteria (memory problems, dependency in ADL, and history of confusion) are part of the standard Hospital Patient Safety Program in the Netherlands [16]. Exclusion criteria were: delirium at admission; laparoscopic cholecystectomy or appendectomy; expected length of stay <48 hours. Patients with hip fracture were not included because they took part in another study that interfered with the aims of this study.

### Ethics Statement

The study was approved by the Medical Ethical Committee (METc) of the University Medical Center Groningen, Groningen, the Netherlands, and was conducted in accordance with the guidelines of the Declaration of Helsinki. In accordance with the Dutch Medical Research (Human Subjects) Act, we did not seek written informed consent from the participants as all data were collected as part of standard patient care. This procedure was approved by the Medical Ethical Committee of the University Medical Center Groningen, Groningen, the Netherlands. The authors BLL and GJI were involved with the collection of the data and had access to identifying information. The data were anonymized prior to analysis.

### Data Collection

All data was collected prospectively by trained research nurses. On hospital admission, medical records were studied for in- and exclusion criteria, reason for admission, illness severity (clinical impression), medical history and current laboratory data. In addition, the Acute Physiology and Chronic Health Evaluation (APACHE) II score was calculated [17]. Patients were interviewed within two days of admission to collect data on physical, cognitive and psychological function before admission. This interview included questions contained in the Groningen Frailty Indicator (GFI)[18]. Type of surgery was ascertained from the patient's medical record.

### Delirium Assessment And Definition

The incidence of postoperative delirium was determined prospectively. The Delirium Observation Screening (DOS) scale was used to screen for delirium [19],[20]. The DOS scale was developed to assess symptoms of delirium based on observations during regular nursing care and can be used as a screening tool as well as a measure of severity of delirium [21]. It is part of the standard Hospital Patient Safety Program in the Netherlands [16]. The DOS scale includes 13-items and was administered by regular ward nurses once per shift (day, evening, and night). The lowest score is 0 points (normal behavior), the highest score is 13 points (strongly altered behavior). The cut-off point is usually set at 3 points with a score ≥3 points indicating delirium (negative predictive value, 99–100%; positive predictive value, 47–89%)[20],[22]. In this study, patients with a score ≥3 points were visited on the same day by a geriatrician for further investigation. The geriatrician evaluated the presence or absence of delirium according to the criteria of the Diagnostic and Statistical Manual of Mental Disorders, fourth edition, text revision (DSM-IV-TR): 1. disturbance of consciousness with reduced ability to focus, sustain, or shift attention; 2. a change in cognition or the development of a perceptual disturbance that is not better accounted for by a preexisting, established, or evolving dementia; 3. the disturbance develops over a short period of time (usually hours to days) and tends to fluctuate during the course of the day; 4. There is evidence from the history, physical examination, or laboratory findings that the disturbance is caused by a medical condition, substance intoxication, or medication side effect [23].

### Risk Stratification Instruments

**Figure 1:**
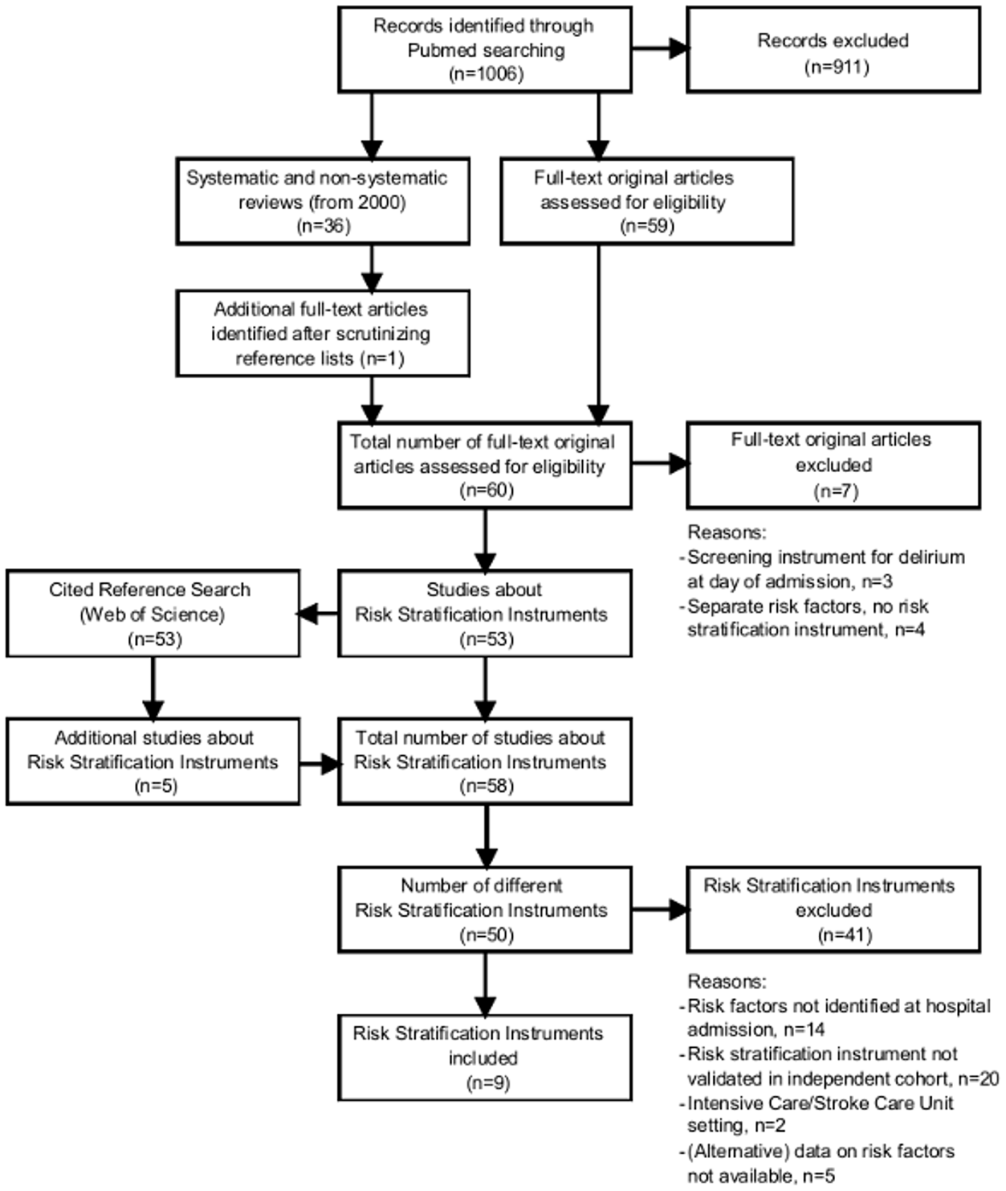
Flow diagram of the different phases of the review. For details of the excluded studies, see Text S1.

A literature search was performed to identify relevant risk stratification instruments for delirium in adult hospital patients (Figure 1). First, MEDLINE/PubMed (1966 to July 2014) was seached with the key concepts “delirium”AND “risk factor”. From this search, we retrieved all potentially relevant original articles published in English since 1966 if the abstract suggested the development of a risk stratification instrument for delirium, and all systematic and non-systematic reviews published in English since January 2000 if the abstract included a description of risk factors for delirium. The reviews were used to identify additional potentially relevant original articles by careful scanning of the texts and reference lists by one of the authors (CJJ). This yielded a total number of 60 original articles. Of these, seven articles were excluded after examination of the full-text version because they did not include the description of a risk stratification instrument. The remaining 53 articles were carefully read and included in a cited reference search using Web of Science (Thomson Reuters, New York, NY) which yielded another five original studies. Thus, in total, we found 58 original studies about risk stratification instruments for delirium. For the present analysis, we included studies if they described a risk stratification instrument for delirium that was developed for practicing clinicians and based on patient characteristics that are commonly identified at hospital admission, and if the risk stratification instrument was validated in at least one independent cohort. Studies were excluded if the risk stratification instrument was highly specific for one type of patient such as, for example, patients in Intensive Care or Stroke units, or if (alternative) data on risk factors was not available. Eventually, nine risk stratification instruments were included [5]–[15]: four were developed in medical patients [5],[8],[14],[15], two in noncardiac surgery patients [6],[11], one in medical and noncardiac surgery patients [7], one in cardiac surgery patients [13], and one in patients with elective arthroplasty or hip fracture [9],[10].

### Application Of The Risk Stratification Instruments

The risk stratification instruments were applied retrospectively to the study population. Although the risk stratification instruments included in this study are based on common risk factors for delirium, the definition and assessment of the risk factors vary widely between the risk stratification instruments (Table S1). For example, cognitive impairment is defined as Mini-Mental State Examination (MMSE) score <24 points by Inouye *et al.* [5], and as Blessed Dementia Rating Scale (BDRS) score ≥4 points by O'Keeffe *et al.* [8]. Similarly, alcohol abuse is defined as Short Michigan Alcoholism Screening Test (SMAST) score >1 point by Pompei *et al.* [7], and as alcohol ≥3 times per week by Freter *et al.* [9],[10]. As a result, some data required for an exact implementation of the original risk stratification instruments was not available. Therefore, some definitions of risk factors were substituted with alternative definitions involving data that was available (Table S1).

### Cut-Off Points For High Risk

For the risk stratification instruments of Inouye (1993), Marcantonio (1994), Pompei (1994), Martinez (2012), and Kobayashi (2013) the cut-off point to identify persons at high risk of postoperative delirium was set at a score of ≥3 points, ≥3 points, ≥8 points, ≥1 point, and at high or quit high risk, respectively (as advised by the authors) [5]–[7],[14],[15]. For the risk stratification instruments of O'Keeffe (1996), Freter (2005), Greene (2009) and Rudolph (2009), we defined the cut-off point for high risk as ≥1 point, ≥2 points, ≥2 points and ≥1 point, respectively. For these risk stratification instruments, the authors did not propose cut-off points. However, the cut-off points that we defined identified patients in whom the risk of postoperative delirium was at least 25% in the original studies [8]–[11],[13]. This was comparable to the risk of postoperative delirium in the high risk groups that were identified by the other risk stratification instruments.

### Statistical Analyses

Normally distributed data are presented as mean and standard deviation (SD). Nonnormally distributed data are presented as median and interquartile range (IQR). The incidence rate of postoperative delirium was calculated per risk score per risk stratification instrument. The 95% confidence intervals (CI) of the incidence rates were calculated as advised by NewCombe and Altman because the absolute number of incident cases was low [24]. Then, we calculated sensitivity and specificity and used receiver operating characteristic (ROC) curves to evaluate the predictive validity of each risk stratification instrument. In ROC curves, an area under the curve (AUC) between 0.50 and 1.00 indicates that the risk stratification instrument performs better than chance. In addition, we calculated the positive and negative predictive value in our study population. Positive agreement (the percentage of patients identified as being at high risk by two different risk stratification instruments) was calculated as advised by Cicchetti and Feinstein [25]; the 95% confidence intervals of positive agreement were calculated as advised by Mckinnon [26]. The level of statistical significance was set at 0.05. All analyses were performed using IBM SPSS Statistics 20.0 (IBM, Armonk, NY).

### Sensitivity Analyses

Because the study population was relatively young, we repeated the analyses in a subsample of older patients (≥60 years). We also repeated the analyses where possible with other definitions of the risk factors to investigate whether the results were dependent on the (possibly arbitrary) definition of the risk factors. This was done because some of the risk factors could not be implemented exactly as described in the original studies. Sensitivity analyses could be done for comorbidity, dependency in activities of daily living (ADL), and impairment in executive function.

## Results

### Study Population

The study population included a total of 292 patients of whom 60% were men (Table 1). Their mean age (SD) was 66 (8) years; 75 percent was aged ≥60 years and 31 percent ≥70 years. Most patients (90%) underwent an elective surgical procedure, either for oncological or benign diagnosis. Seventy-two percent of the participants had two or more comorbidities and 51% used four or more medications (Table 1). The incidence of postoperative delirium was nine percent (95%CI, 6–13%).

**Table 1:** Characteristics of the study population.

All, n (%)		292	(100)
Gender, n (%)	Men	175	(60)
	Women	117	(40)
Age, mean (SD), years		66	(8)
Age groups, n (%), years	50–59	75	(26)^a^
	60–69	128	(44)
	70–79	69	(24)
	≥80	20	(7)
APACHE II, median (IQR), points		5	(4–7)
Comorbidities, n (%)	0–1 comorbidities	82	(28)
	≥2 comorbidities	210	(72)
	Diabetes mellitus	54	(19)
	Hypertension	109	(37)
	Other cardiovascular disease	87	(30)
	Cerebrovascular disease	28	(10)
	Other neurological disease	12	(4)
	Chronic pulmonary disease	35	(12)
	Chronic renal disease	20	(7)
Medication use, n (%)	0–3 medications	144	(49)
	≥4 medications	148	(51)
History of delirium, n (%)		41	(14)
Cognitive impairment, n (%)		40	(14)
Admission type, n (%)	Elective	264	(90)
	Emergency	28	(10)
Type of surgery, n (%)	Oncologic surgery	93	(32)
	General surgery	84	(29)
	Vascular surgery	54	(18)
	Hepatobiliary surgery	39	(13)
	Other	22	(8)
Length of stay, median (IQR), days		8	(4–14)
ICU admission, n (%)		81	(28)
Postoperative delirium, n (%)		25	(9)

Abbreviations: APACHE, Acute Physiology and Chronic Health Evaluation; ICU, intensive care unit; IQR, interquartile range; SD, standard deviation.

a Sum of percentages not equal to 100 due to rounding.

### Content Of Risk Stratification Instruments

The nine risk stratification instruments comprised many different risk factors (Table 2). The number of risk factors per risk stratification instrument varied between two and six. Many risk factors were included in several risk stratification instruments. The most common risk factors were cognitive impairment (in seven risk stratification instruments), high age (in four risk stratification instruments), and alcohol abuse and dependency in activities of daily living (in three risk stratification instruments). In our study population, there were large differences between the risk stratification instruments as well as within the risk stratification instruments in the prevalence of the risk factors (Table 2). For example, the risk stratification instrument of Greene (2009) comprised two risk factors with a prevalence rate of 30% whereas the three risk factors included by the risk stratification instrument of Martinez (2012) had a prevalence rate between one and five percent.

**Table 2:** Risk factors included by the risk stratification instruments for postoperative delirium.

Risk stratification instrument (first author, year of publication)	Risk factor^a^	Prevalence	
		n/N^b^	(%)
Inouye, 1993	Cognitive impairment	40/292	(14)
	Vision impairment	16/291	(6)
	APACHE II >16 points	3/292	(1)
	Blood urea nitrogen/creatinine ratio (in mg/dL) ≥18 at admission	165/280	(59)
Marcantonio, 1994	Cognitive impairment	40/292	(14)
	Age ≥70 year	89/292	(30)
	Alcohol abuse or addiction	39/292	(13)
	Low physical fitness	104/291	(36)
	Preoperative sodium level <130 or>150 mmol/L; or potassium level <3.0 or >6.0 mmol/L	3/280	(1)
	Surgery for aortic aneurysm	16/292	(5)
	Noncardiac thoracic surgery	20/292	(7)
	Other noncardiac surgery	256/292	(88)
Pompei, 1994	Cognitive impairment	40/292	(14)
	Alcohol abuse or addiction	39/292	(13)
	Recently feeling downhearted or sad	92/290	(32)
	Comorbidity, ≥2 diseases	210/292	(72)
O'Keeffe, 1996	Cognitive impairment	40/292	(14)
	Severe illness (subjective judgement)	2/283	(1)
	Urea level >10 mmol/L at admission	27/281	(10)
Freter, 2005	Cognitive impairment or previous delirium	74/292	(25)
	Age ≥80 year	20/292	(7)
	Alcohol abuse or benzodiazepine use	70/292	(24)
	Vision or hearing impairment	46/291	(16)
	Unable to walk outside, to (un)dress or to go to the toilet without help	22/291	(8)
Greene, 2009	Letter fluency (initial letter S), ≤5 words/min^c^	30/89	(33)
	Recently feeling downhearted or sad	92/290	(32)
Rudolph, 2009	Cognitive impairment	40/292	(14)
	History of cerebrovascular disease	28/292	(10)
	Recently feeling downhearted or sad	92/290	(32)
	Preoperative albumin level <3.6 or>4.4 g/dL	67/175	(38)
Martinez, 2012	Age ≥86 year	4/292	(1)
	Use of psychotropic drugs	14/292	(5)
	Unable to walk outside, to (un)dress or to go to the toilet without help (at least 2 out of 3)	9/291	(3)
Kobayashi, 2013	History of confusion	41/292	(14%)
	Age ≤50 year	3/292	(1%)
	Age 51–75 year	249/292	(85%)
	Age>75 year	40/292	(14%)
	Unable to shop, to walk outside, to (un)dress, to go to the toilet without help, or cognitive impairment	68/291	(23%)
	Malignancy	141/292	(48%)

Abbreviation: APACHE, Acute Physiology and Chronic Health Evaluation.

a The definition of some risk factors differed from their definition in the original studies (see Table S1).

b For some variables, N<292 due to missing data.

c Letter fluency was measured in a subset of patients.

### Predictive Performance

**Figure 2:**
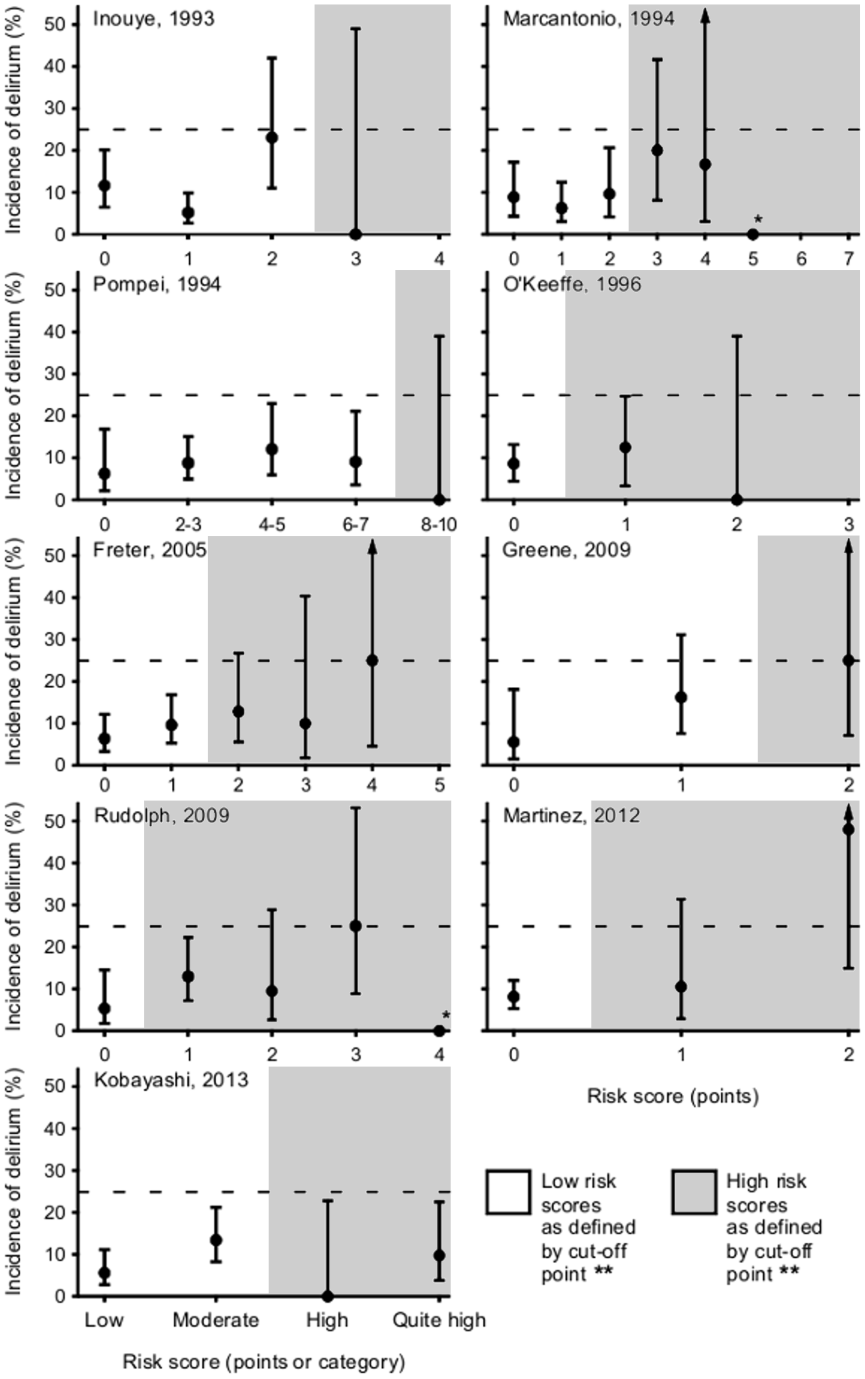
Observed incidence rate of postoperative delirium by risk stratification instrument (first author, year of publication) and risk score. Bars represent 95% confidence intervals. Dashed lines correspond to an incidence rate of 25%. * 95% confidence interval omitted because category included only one person. ** For the risk stratification instruments of Inouye (1993), Marcantonio (1994), Pompei (1994), Martinez (2012), and Kobayashi (2013) the cut-off point was defined by the authors of the original study. For the definition of the cut-off points of the other risk stratification instruments, see text. For the number of persons per risk score, see Table S2.

The highest observed incidence of postoperative delirium for any risk stratification instrument and risk score was 50% (95%CI, 15–85%) which was found for patients with two points according to the risk stratification instrument of Martinez (2012) [14]. However, for eight risk stratification instruments, the highest observed incidence rate of postoperative delirium per risk score was equal to or less than 25% (Figure 2). In addition, some risk stratification instruments did not show a clear association between observed incidence of postoperative delirium and risk score (Figure 2).

**Figure 3:**
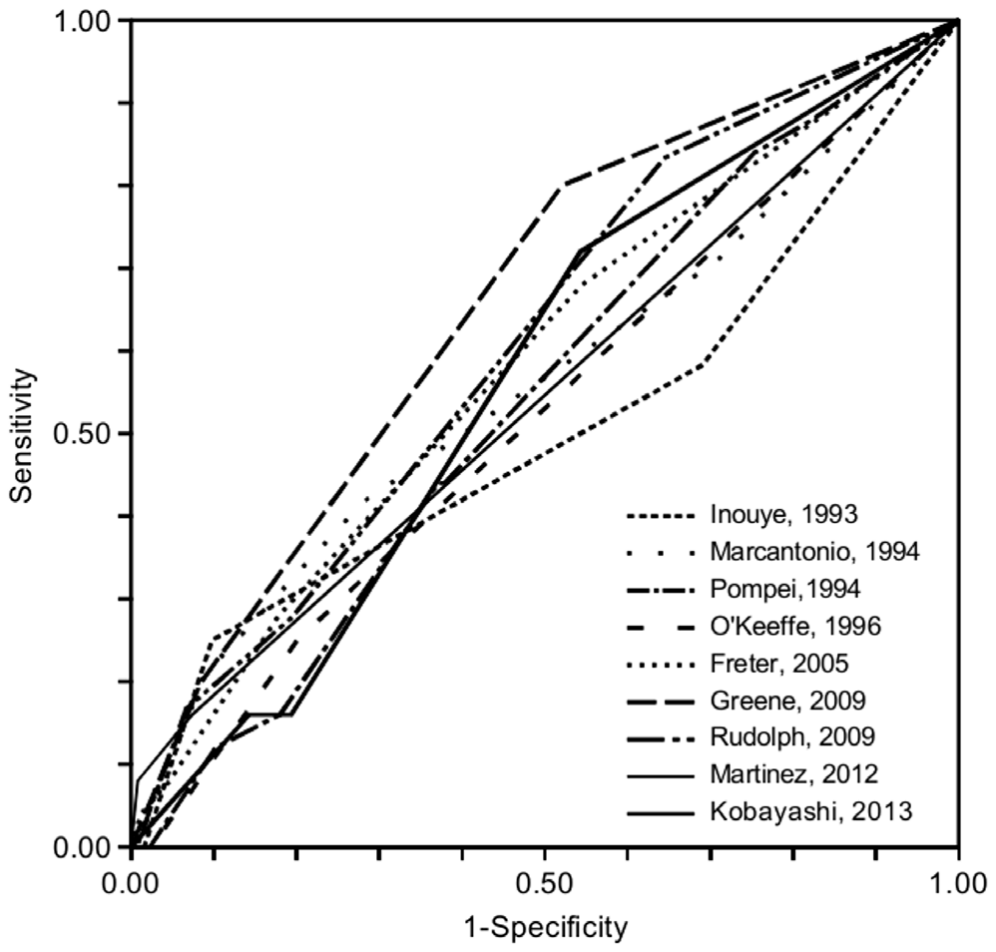
Receiver operating characteristic (ROC) curves of the risk stratification instruments for postoperative delirium (first author, year of publication). For all risk stratification instruments, the area under the curve (AUC) was not statistically different from 0.50 (for details, see Table 3).

ROC curve analysis showed that the risk stratification instruments did not predict postoperative delirium better than chance (Figure 3). For all risk stratification instruments, the AUC was not statistically different from 0.50 (Table 3). If the outcomes of the risk stratification instruments were dichotomized into being at low vs. high risk of postoperative delirium, the positive predictive values of the risk stratification instruments were between 0% and 25% and the negative predictive values between 89% and 95% (Table 3).

**Table 3:** Performance of the risk stratification instruments in identifying patients at high risk for postoperative delirium.^a^

Risk stratification instrument (first author, year of publication)	Sensitivity (%)	Specificity (%)	Positive predictive value (%)	Negative predictive value (%)	ROC analysis	
					AUC (95%CI)	*p*-value
Inouye, 1993	0	98	0	91	0.50 (0.36–0.64)	0.97
Marcantonio, 1994	21	91	19	92	0.56 (0.43–0.69)	0.35
Pompei, 1994	0	98	0	91	0.54 (0.43–0.65)	0.49
O'Keeffe, 1996	25	80	11	91	0.52 (0.40–0.64)	0.73
Freter, 2005	28	82	13	92	0.58 (0.47–0.70)	0.17
Greene, 2009	20	92	25	89	0.66 (0.48–0.83)	0.11
Rudolph, 2009	83	36	14	95	0.61 (0.48–0.77)	0.13
Martinez, 2012	16	93	17	92	0.55 (0.42–0.67)	0.45
Kobayashi, 2013	16	81	7	91	0.57 (0.46–0.67)	0.27

Abbreviations: AUC: area under the curve; CI: confidence interval; ROC: receiver operating characteristic.

a For the cut-off points of the risk stratification instruments for low vs. high risk, see text.

### Agreement

The positive agreement between the risk stratification instruments varied between 0 and 57% (95%CI, 26–88%). On average, the risk stratification instruments of Inouye (1993) and Pompei (1994) showed the lowest positive agreement with other risk stratification instruments (Table S3).

### Sensitivity Analyses

The analyses yielded essentially similar results when they were repeated in patients aged ≥60 years (mean age, 69; SD, 7 years). The incidence of postoperative delirium in this age group was 10 percent (95%CI, 7–15%). It was found for all risk stratification instruments that the test characterictics in persons aged ≥60 years were comparable to the test characteristics in persons aged ≥50 years (Text S2, Table A). The performance of the risk stratification instruments was also essentially similar for different definitions of comorbidity (risk stratification instrument of Pompei, 1994), ADL (risk stratification instruments of Freter, 2005; Martinez 2012; Kobayashi, 2013), and for different definitions of executive function (risk stratification instrument of Greene, 2009) (Text S2, Table B-D).

## Discussion

Reliable prediction of postoperative delirium is essential for the planning of good peroperative care in older persons. If it is recognized early that an older surgical patient is at increased risk of postoperative delirium, it is possible to select and tailor interventions that may prevent delirium [4], and to inform a patient properly about the risks of surgery. However, in this study, we found that commonly used risk stratification instruments performed no better than chance in distinguishing between patients at low or high risk of postoperative delirium. Accordingly, the positive predictive value of the risk stratification instruments was poor. Also, the agreement between the risk stratification instruments in identifying patients at high risk of postoperative delirium (positive agreement) was low. Therefore, the generalizability of these commonly used risk stratification instruments is probably limited.

All risk stratification instruments that were investigated in this study were previously evaluated in at least one independent validation sample. Most risk stratification instruments were developed and evaluated in studies that included a development and independent validation sample from the same target population [5]–[8],[13],[14]. Other risk stratification instruments were developed and evaluated in separate studies that included different categories of patients [9]–[12], such as, for example, patients undergoing elective hip or knee arthroplasty, or patients with hip fracture [9],[10]. Nonetheless, most risk stratification instruments performed far better in the original studies than in this study. Whereas several original studies reported positive predictive values between 40% and 100% [6]–[8],[10],[11],[13],[14], this study found positive predictive values that were only between 0% and 25%. Thus, the risk stratification instruments yielded highly divergent results in different patient populations.

The large differences in performance of the risk stratification instruments could be ascribed to several factors. First, there was a difference between our study and the original studies in the definition and assessment of a number of risk factors included by the risk stratification instruments. This was due to the unavailability of some data required for the exact implementation of the risk stratification instruments. Although this could have influenced some of the results, the effect is likely to be small if it is assumed that the risk stratification instruments are robust. Second, there were differences in the incidence rate of delirium between the original development and validation studies. In most original studies, the incidence of delirium was between 15% and 52% [7],[13]. Thus, compared to these incidence rates, the incidence of delirium in the current study (9%) was relatively low. Third, some of the risk stratification instruments were developed in medical patients [5],[8],[14],[15], whereas the current study involved surgical patients. Fourth, several risk stratification instruments were developed in patient populations that were considerably older than the patient population of the current study [5],[7]–[10],[14],[15]. On the other hand, all risk stratification instruments were based on the same conceptual model that is widely accepted among experts in the field. In this conceptual model, the onset of delirium is not caused by one single factor but the outcome of a complex interaction of various risk factors [4]. Many of these risk factors have been identified and are included in the risk stratification instruments that were investigated in this study. Consequentially, it is not likely that the performance of these risk stratification instruments is strongly dependent on the characteristics of a specific study population.

To our knowledge, this is the first study that included data on agreement between risk stratification instruments for (postoperative) delirium. Interestingly, it was found that for most risk stratification instruments, positive agreement was very low. This implies that the various risk stratification instruments identified very different patients as being at high risk for postoperative delirium. This low positive agreement was somewhat surprising as the risk stratification instruments shared various risk factors such as, for example, older age, cognitive impairment, alcohol abuse and visual or hearing impairment, that are established risk factors for delirium [4]. It is unlikely that the low positive agreement is due to a different definition and assessment of these risk factors in the distinct risk stratification instruments as in this study, their definition and assessment was very similar. Therefore, the low positive agreement might be due to differences between the risk stratification instruments in the combination of risk factors although in our opinion, this would point to a certain lack of robustness of the commonly accepted risk factors for delirium. A more likely explanation is that the etiology of (postoperative) delirium is far more complex than currently understood and that probably, important risk factors have yet to be discovered. Although the concept of predisposing and precipitating risk factors is widely accepted [4], the common risk stratification instruments are mainly based on predisposing risk factors only. Possibly, predictive performance and agreement of the risk stratification instruments could be improved by adding clearly defined and quantifiable precipitating risk factors that are part of anesthetic and surgical procedures.

The positive predictive value is probably the most important test characteristic of a risk stratification instrument for postoperative delirium as the incidence rate of postoperative delirium may be relatively low. In this study, the differences in test characteristics between most risk stratification instruments were small but the best positive predictive value was found for the risk stratification instruments of Greene (2009) and Marcantonio (1994). In our opinion, these risk stratification instruments are equally easy to use in clinical practice.

Some limitations of our study have to be discussed. First, as discussed above, a number of risk factors was defined differently compared to the original studies. This was most clear for cognitive impairment that was defined by the performance on a formal screening test in some studies [5],[6], and by the positive answer to only one question in our study. However, in our opinion, this is not a sufficient explanation for the low performance of the risk stratification instruments because there are also differences between the original studies in the definition of risk factors. For example, cognitive impairment was defined as MMSE score <24 points in the study by Inouye *et al.* [5], as cognitive status interfering with social functioning in the study by O'Keeffe *et al.* [8], and as MMSE score <24 points or previous postoperative delirium in the studies by Freter *et al.* [9],[10]. Second, the observed incidence of postoperative delirium was relatively low. Although some cases of delirium could have been missed, this is unlikely as the DOS scale was used and this scale has a high negative predictive value for delirium [20],[22]. Moreover, the incidence of postoperative delirium in this study was comparable to that in some of the original studies [6],[9],[11]. Third, the risk stratification instruments were applied retrospectively. Although this could have caused some errors in the risk stratification of individual patients, we think that this effect is small because all data used for the application of the risk stratification instruments was collected prospectively. Fourth, some risk stratification instruments were not developed in surgical patients but in medical patients. However, it is not feasible for clinicians to use different risk stratification instruments for different types of patients. Therefore, most clinicians use the risk stratification instrument of their choice for every kind of patient.

Our study also has several strengths. First, the study sample included consecutive patients from diverse surgical specialties. Second, all data was collected prospectively. Third, all patients were routinely screened for delirium with the DOS scale which has a high negative predictive value, and if the screening was positive, patients were further investigated by an expert geriatrician. Fourth, and most importantly, our study comprised a study population that was wholly independent from the development and validation samples of the original studies.

In conclusion, in this independent validation cohort, the performance and agreement of commonly used risk stratification instruments for (postoperative) delirium were poor. However, the translation of these findings into clinical practice requires some caution because the implementation of the risk stratification instruments in this study was not exactly similar to the implementation in the original studies. Nevertheless, we think that the usefulness of the current risk stratification instruments for delirium can be questioned and that these instruments need more rigorous evaluation in well designed prospective studies that include different clinical settings and patient populations.

## Supporting Information

Table S1 **Definition of risk factors included by the risk stratification instruments for postoperative delirium.**
(DOC)

Table S2 **Number of persons per risk category per risk stratification instrument.**
(DOC)

Table S3 **Positive agreement between the risk stratification instruments for postoperative delirium.**
(DOC)

Text S1 **Excluded articles and risk stratification instruments.**
(DOC)

Text S2 **Sensitivity analyses.**
(DOC)

## References

[1] Etzioni DA, Liu JH, Maggard MA, Ko CY (2003) The aging population and its impact on the surgery workforce. Ann Surg 238:170–177. 12894008 10.1097/01.SLA.0000081085.98792.3d PMC1422682

[2] Brooks Carthon JM, Jarrin O, Sloane D, Kutney-Lee A (2013) Variations in postoperative complications according to race, ethnicity, and sex in older adults. J Am Geriatr Soc 61:1499–1507. 24006851 10.1111/jgs.12419 PMC3773274

[3] Chow WB, Rosenthal RA, Merkow RP, Ko CY, Esnaola NF, et al (2012) Optimal preoperative assessment of the geriatric surgical patient: a best practices guideline from the American College of Surgeons National Surgical Quality Improvement Program and the American Geriatrics Society. J Am Coll Surg 215:453–466. 22917646 10.1016/j.jamcollsurg.2012.06.017

[4] Inouye SK, Westendorp RG, Saczynski JS (2014) Delirium in elderly people. Lancet 383:911–922. 23992774 10.1016/S0140-6736(13)60688-1 PMC4120864

[5] Inouye SK, Viscoli CM, Horwitz RI, Hurst LD, Tinetti ME (1993) A predictive model for delirium in hospitalized elderly medical patients based on admission characteristics. Ann Intern Med 119:474–481. 8357112 10.7326/0003-4819-119-6-199309150-00005

[6] Marcantonio ER, Goldman L, Mangione CM, Ludwig LE, Muraca B, et al (1994) A clinical prediction rule for delirium after elective noncardiac surgery. JAMA 271:134–139. 8264068

[7] Pompei P, Foreman M, Rudberg MA, Inouye SK, Braund V, et al (1994) Delirium in hospitalized older persons: outcomes and predictors. J Am Geriatr Soc 42:809–815. 8046190 10.1111/j.1532-5415.1994.tb06551.x

[8] O'Keeffe ST, Lavan JN (1996) Predicting delirium in elderly patients: development and validation of a risk-stratification model. Age Ageing 25:317–321. 8831879 10.1093/ageing/25.4.317

[9] Freter SH, Dunbar MJ, MacLeod H, Morrison M, MacKnight C, et al (2005) Predicting post-operative delirium in elective orthopaedic patients: the Delirium Elderly At-Risk (DEAR) instrument. Age Ageing 34:169–171. 15713861 10.1093/ageing/afh245

[10] Freter SH, George J, Dunbar MJ, Morrison M, Macknight C, et al (2005) Prediction of delirium in fractured neck of femur as part of routine preoperative nursing care. Age Ageing 34:387–388. 15955758 10.1093/ageing/afi099

[11] Greene NH, Attix DK, Weldon BC, Smith PJ, McDonagh DL, et al (2009) Measures of executive function and depression identify patients at risk for postoperative delirium. Anesthesiology 110:788–795. 19326494 10.1097/aln.0b013e31819b5ba6 PMC2953946

[12] Smith PJ, Attix DK, Weldon BC, Greene NH, Monk TG (2009) Executive function and depression as independent risk factors for postoperative delirium. Anesthesiology 110:781–787. 19326492 10.1097/aln.0b013e31819b5bc2 PMC2757787

[13] Rudolph JL, Jones RN, Levkoff SE, Rockett C, Inouye SK, et al (2009) Derivation and validation of a preoperative prediction rule for delirium after cardiac surgery. Circulation 119:229–236. 19118253 10.1161/CIRCULATIONAHA.108.795260 PMC2735244

[14] Martinez JA, Belastegui A, Basabe I, Goicoechea X, Aguirre C, et al (2012) Derivation and validation of a clinical prediction rule for delirium in patients admitted to a medical ward: an observational study. BMJ Open 2:e00159910. 10.1136/bmjopen-2012-001599 PMC3467592 22983876

[15] Kobayashi D, Takahashi O, Arioka H, Koga S, Fukui T (2013) A prediction rule for the development of delirium among patients in medical wards: Chi-Square Automatic Interaction Detector (CHAID) decision tree analysis model. Am J Geriatr Psychiatry 21:957–962. 23567433 10.1016/j.jagp.2012.08.009

[16] [Anonymous]. Hospital Patient Safety Program. 2013. Available: http://www.vmszorg.nl/_page/vms_inline?nodeid=4635&subjectid=10977 (accessed 17 February 2014).

[17] Knaus WA, Draper EA, Wagner DP, Zimmerman JE (1985) APACHE II: a severity of disease classification system. Crit Care Med 13:818–829. 3928249

[18] Schuurmans H, Steverink N, Lindenberg S, Frieswijk N, Slaets JP (2004) Old or frail: what tells us more? J Gerontol A Biol Sci Med Sci 59:M962–5. 15472162 10.1093/gerona/59.9.m962

[19] Schuurmans MJ, Shortridge-Baggett LM, Duursma SA (2003) The Delirium Observation Screening Scale: a screening instrument for delirium. Res Theory Nurs Pract 17:31–50. 12751884 10.1891/rtnp.17.1.31.53169

[20] van Gemert LA, Schuurmans MJ (2007) The Neecham Confusion Scale and the Delirium Observation Screening Scale: capacity to discriminate and ease of use in clinical practice. BMC Nurs 6:3. 17394635 10.1186/1472-6955-6-3 PMC1852304

[21] Scheffer AC, van Munster BC, Schuurmans MJ, de Rooij SE (2011) Assessing severity of delirium by the Delirium Observation Screening Scale. Int J Geriatr Psychiatry 26:284–291. 20665557 10.1002/gps.2526

[22] Koster S, Hensens AG, Oosterveld FG, Wijma A, van der Palen J (2009) The delirium observation screening scale recognizes delirium early after cardiac surgery. Eur J Cardiovasc Nurs 8:309–314. 19285452 10.1016/j.ejcnurse.2009.02.006

[23] American Psychiatric Association. (2000) Diagnostic and Statistical Manual of Mental Disorders, Fourth Edition, Text Revision (DSM-IV-TR). Arlington, VA: American Psychiatric Association.

[24] Newcombe RG, Altman DG (2000) Proportions and their differences. In: Altman DG, Machin D, Bryant TN, Gardner MJ, editors. Statistics with confidence. London: BMJ Books. pp. 46–47.

[25] Cicchetti DV, Feinstein AR (1990) High agreement but low kappa: II. Resolving the paradoxes. J Clin Epidemiol 43:551–558. 2189948 10.1016/0895-4356(90)90159-m

[26] Mackinnon A (2000) A spreadsheet for the calculation of comprehensive statistics for the assessment of diagnostic tests and inter-rater agreement. Comput Biol Med 30:127–134. 10758228 10.1016/s0010-4825(00)00006-8

