# Supplementary material for: Performance And Agreement Of Risk Stratification Instruments For Postoperative Delirium In Persons Aged 50 Years Or Older
Source: PLoS One. 2014 Dec 2;9(12):e113946. doi: 10.1371/journal.pone.0113946 (PMC4252072; doi:10.1371/journal.pone.0113946)
Supplement: Table S1 — (DOC) [file pone.0113946.s001.doc]

**Table S1.** Definition of risk factors included by the risk stratification instruments for postoperative delirium.

| Risk stratification instrument (first author, year of publication) | Risk factor | Definition in original study | Definition in this study |
| --- | --- | --- | --- |
| Inouye, 1993 | Cognitive impairment | MMSE <24 points | Patient or a family member answered “yes” to the question “Do you have memory problems?”^a^ |
|  | Vision impairment | Corrected vision <20/70 on binocular test | Patient or a family member answered “yes” to the question “Do you experience problems in daily life due to poor vision?”^b^ |
|  | Illness severity | APACHE II >16 points | As in original study |
|  | Dehydration | BUN-to-creatinine ratio (in mg/dL) ≥18 at admission | As in original study |
| Marcantonio, 1994 | Cognitive impairment | TICS <30 points | Patient or a family member answered “yes” to the question “Do you have memory problems?”^a^ |
|  | Age | <70 vs. ≥70 year | As in original study |
|  | Alcohol abuse | Having a problem with drinking too much alcohol | Alcohol abuse or addiction as reported by the patient or a family member. |
|  | Physical functioning | Unable to walk 4 km/h for one block, or dress themselves without stopping (SAS-IV) | Physical fitness ≤ 6/10 points as rated by the patient when asked the question “What mark do you give yourself for physical fitness?”^c^ |
|  | Abnormal levels of preoperative sodium, potassium or glucose | Sodium <130 or >150 mmol/L; potassium <3.0 or >6.0 mmol/l; glucose <3.3 or >16.7 mmol/L | Sodium <130 or >150 mmol/l; potassium <3.0 or >6.0 mmol/l |
|  | Type of surgery | Surgery for aortic aneurysm or noncardiac thoracic surgery or other noncardiac surgery | As in original study |
| Pompei, 1994 | Cognitive impairment | For education <high school, MMSE <21 points; for high school education, MMSE <23 points; for college education, MMSE <24 points | Patient or a family member answered “yes” to the question “Do you have memory problems?”^a^ |
|  | Alcohol abuse | SMAST >1 points, or CAGE >1 point | Alcohol abuse or addiction as reported by the patient or a family member. |
|  | Depression | Development cohort, GDS-15 >4 points; validation cohort, GDS >6points. | Patient or a family member answered “yes” or “sometimes” to the question “Did you feel downhearted or sad recently?”^d^ |
|  | Comorbidity | Major Diagnostic Categories (MDC) >3 points | ≥2 diseases as documented in the patient’s medical record. |
| O’Keeffe, 1996 | Cognitive impairment | Cognitive status interfering with social functioning, or BDRS ≥4 points | If patient or a family member answered “yes” to the question “Do you have memory problems?”^a^ |
|  | Illness severity | Subjective rating by a physician | As in original study |
|  | Abnormal level of urea | Blood urea >10 mmol/L | As in original study |
| Freter, 2005 | Cognitive impairment | MMSE <24 points, or previous postoperative delirium | Patient or a family member answered “yes” to the question “Do you have memory problems?”^a^, or patient or a family member answered “yes” to the question “Did you feel confused during previous illness or hospitalization?”^e^ |
|  | Age | <80 vs. ≥80 year | As in original study |
|  | Substance abuse | Alcohol or benzodiazepines more than 3 times a week | Alcohol abuse or addiction or use of benzodiazepines as reported by the patient or a family member. |
|  | Vision or hearing impairment | Using hearing aid of having very poor vision | Patient or a family member answered “yes” to the question “Do you experience problems in daily life due to poor vision?”^b^, or “Do you experience problems in daily life due to poor hearing?”^f^ |
|  | Dependency in activities of daily life | Needing assistance in bathing, dressing, toileting, grooming or feeding | Patient or a family member answered “no” to the question “Are you able to walk around outside without help?”^g^, or “Are you able to dress and undress without help?”^h^, or “Are you able to go to the toilet without help?”^i^ |
| Greene, 2009 | Impairment in executive function | TMT-B >154 seconds, or MSIT ≥60 seconds | Letterfluency (initial letter S) ≤5 words/min |
|  | Depression | GDS-15 >4 points, or BDI ≥14 points | Patient or a family member answered “yes” or “sometimes” to the question “Did you feel downhearted or sad recently?”^d^ |
| Rudolph, 2009 | Cognitive impairment | MMSE ≤23 points and MMSE 24-27 points as compared to MMSE 28-30 points | Patient or family member answered “yes” to the question “Do you have memory problems?”^a^ |
|  | History of stroke or TIA | History of stroke or transient ischemic attack | History of cerebrovascular disease as documented in the patient’s medical record. |
|  | Depression | GDS-15 >4 points | Patient or a family member answered “yes” or “sometimes” to the question “Did you feel downhearted or sad recently?”^d^ |
|  | Abnormal preoperative level of albumin | Serum albumin <3.6 or >4.4 g/dL | As in original study |
| Martinez, 2012 | Age | <86 vs. ≥ 86 year | As in original study |
|  | Use of psychotropic drugs | Use of benzodiazepines, antidepressants, antidementia drugs or antipsychotics | As in original study |
|  | Dependency in activities of daily life | Dependency in five or more (out of six) activities of daily living: personal hygiene, (un)dressing, toileting, ambulation, bowel and bladder control, feeding. | Patient or a family member answered “no” to at least two of the questions “Are you able to walk around outside without help?”^g^, or “Are you able to dress and undress without help?”^h^, or “Are you able to go to the toilet without help?”^i^ |
| Kobayashi, 2013 | History of confusion | Delirium history | Patient or a family member answered “yes” to the question “Did you have memory problems or feel confused during previous illness or hospitalization?”^e^ |
|  | Impairment in ADL | Not completely independent according to FIM | Patient or a family member answered “no” to at least one of the questions “Are you able to shop”^j^, “Are you able to walk around outside without help?”^g^, “Are you able to dress and undress without help?”^h^, or “Are you able to go to the toilet without help?”^i^, or answered “yes” to the question “Do you have memory problems?”^a^ |
|  | Age | ≤75 vs. >75 year | As in original study |
|  | Malignancy | Any kind of malignancy (treated malignancy not included) | As in original study |

Abbreviations: APACHE II, Acute Physiology and Chronic Health Evaluation II [1]; BDI, Beck Depression Inventory [2]; BDRS, Blessed Dementia Rating Scale [3]; BUN, blood urea nitrogen; CAGE, Cutting down, Annoyance by criticism, Guilty feeling, and Eye-openers [4]; FIM, Functional Independence Measure [5]; GDS-15, Geriatric Depression Scale, 15 items version [6]; MDC, Major Diagnostic Categories [7];MMSE, Mini-Mental State Examination [8]; MSIT, Modified Stroop Interference Task [9]; SAS, Specific Activity Scale [10]; SMAST, Short Michigan Alcoholism Screening Test [11]; TICS, Telephone Interview for Cognitive status [12]; TMT-B, Trail Making Test, part B [13].

1. From the Dutch Hospital Safety Management Program, theme Vulnerable Elderly [14]: screening questionnaire for risk factors for delirium, question 1.
2. From the Groningen Frailty Indicator [15]: item 6.
3. From the Groningen Frailty Indicator [15]: item 5.
4. From the Groningen Frailty Indicator [15]: item 14.
5. From the Dutch Hospital Safety Management Program, theme Vulnerable Elderly [13]: screening questionnaire for risk factors for delirium, question 3.
6. From the Groningen Frailty Indicator [15]: item 7.
7. From the Groningen Frailty Indicator [15]: item 2.
8. From the Groningen Frailty Indicator [15]: item 3.
9. From the Groningen Frailty Indicator [15]: item 4.
10. From the Groningen Frailty Indicator [15]: item 1.

**References**

1. Knaus WA, Draper EA, Wagner DP, Zimmerman JE. APACHE II: a severity of disease classification system. Crit Care Med. 1985;13:818-829.
2. Beck AT, Ward CH, Mendelsohn M, Mock J, Erbaugh J. An inventory for measuring depression. Arch Gen Psychiatry. 1961;4:561–571.
3. Blessed G, Tomlinson BE, Roth M. The association between quantitative measures of dementia and of senile change in the cerebral grey matter of elderly subjects. Br J Psychiatry. 1968;114:797-811.
4. Ewing JA. Detecting alcoholism. The CAGE questionnaire. JAMA. 1984;252:1905-1907.
5. Granger CV, Hamilton BB, Linacre JM, Heinemann AW, Wright BD. Performance profiles of the functional independence measure Am J Phys Med Rehabil. 1993;72:84-89.
6. Sheikh JI, Yesavage JA. Geriatric Depression Scale (GDS): recent evidence and development of a shorter version. In: Brink TL (Ed.). Clinical gerontology: a guide to assessment and intervention. New York: Haworth Press, 1986, pp. 165-173.
7. Fetter RB, Shin Y, Freeman JL, Averill RF, Thompson JD. Case mix definition by diagnosis-related groups.Med Care. 1980;18 (2 Suppl):iii, 1-53.
8. Folstein MF, Folstein SE, McHugh PR. "Mini-mental state". A practical method for grading the cognitive state of patients for the clinician. J Psychiatr Res. 1975;12:189-198.
9. Bohnen N, Twijnstra A, Jolles J. Performance in the Stroop color word test in relationship to the persistence of symptoms following mild head injury. Acta Neurol Scand. 1992;85:116–121.
10. Goldman L, Hashimoto B, Cook EF, Loscalzo A. Comparative reproducibility and validity of systems for assessing cardiovascular functional class: advantages of a new specific activity scale. Circulation. 1981;64:1227-1234.
11. Selzer ML, Vinokur A, van Rooijan L. A self-administered Short Michigan Alcoholism Screening Test (SMAST). J Stud Alcohol. 1975;36:117-126.
12. Brandt J, Spencer M, Folstein M. The Telephone Interview for Cognitive Status. Neuropsychiatry Neuropsychol Behav Neurol. 1988;1:111-117.
13. Reitan RM. The relation of the trail making test to organic brain damage. J Consult Psychol. 1955; 19:393-394.
14. Dutch Hospital Safety Management Program. 2013. http://www.vmszorg.nl/_page/vms_inline?nodeid=4635&subjectid=10977 (accessed 17 February 2014).
15. Schuurmans H, Steverink N, Lindenberg S, Frieswijk N, Slaets JP. Old or frail: what tells us more? J Gerontol A Biol Sci Med Sci. 2004;59:M962-5.
