# Supplementary material for: Performance And Agreement Of Risk Stratification Instruments For Postoperative Delirium In Persons Aged 50 Years Or Older
Source: PLoS One. 2014 Dec 2;9(12):e113946. doi: 10.1371/journal.pone.0113946 (PMC4252072; doi:10.1371/journal.pone.0113946)
Supplement: Table S2 — (DOC) [file pone.0113946.s002.doc]

**Table S2.** Number of persons per risk category per risk stratification instrument.

| **Risk stratification instrument (first author, year of publication)** | | **Risk Category** | | | | | | | | **All, n^a^** |
| --- | --- | --- | --- | --- | --- | --- | --- | --- | --- | --- |
| Inouye, 1993 | Risk score (points) | 0 | 1 | 2 | 3 | 4 | N/A | N/A | N/A |  |
|  | N_total_ | 86 | 154 | 26 | 4 | 0 | - | - | - | 270 |
|  | Delirium, n | 10 | 8 | 6 | 0 | 0 | - | - | - | 24 |
| Marcantonio, 1994 | Risk score (points) | 0 | 1 | 2 | 3 | 4 | 5 | 6 | 7 |  |
|  | N_total_ | 79 | 112 | 52 | 20 | 6 | 1 | 0 | 0 | 270 |
|  | Delirium, n | 7 | 7 | 5 | 4 | 1 | 0 | 0 | 0 | 24 |
| Pompei, 1994 | Risk score (points) | 0 | 2-3 | 4-5 | 6-7 | 8-10 | N/A | N/A | N/A |  |
|  | N_total_ | 48 | 125 | 58 | 44 | 6 | - | - | - | 281 |
|  | Delirium, n | 3 | 11 | 7 | 4 | 0 | - | - | - | 25 |
| O’Keeffe, 1996 | Risk score (points) | 0 | 1 | 2 | 3 | N/A | N/A | N/A | N/A |  |
|  | N_total_ | 209 | 48 | 6 | 0 | - | - | - | - | 263 |
|  | Delirium, n | 18 | 6 | 0 | 0 | - | - | - | - | 24 |
| Freter, 2005 | Risk score (points) | 0 | 1 | 2 | 3 | 4 | 5 | N/A | N/A |  |
|  | N_total_ | 125 | 104 | 39 | 10 | 4 | 0 | - | - | 282 |
|  | Delirium, n | 8 | 10 | 5 | 1 | 1 | 0 | - | - | 25 |
| Greene, 2009 | Risk score (points) | 0 | 1 | 2 | N/A | N/A | N/A | N/A | N/A |  |
|  | N_total_ | 36 | 37 | 8 | - | - | - | - | - | 81 |
|  | Delirium, n | 2 | 6 | 2 | - | - | - | - | - | 10 |
| Rudolph, 2009 | Risk score (points) | 0 | 1 | 2 | 3 | 4 | N/A | N/A | N/A |  |
|  | N_total_ | 56 | 77 | 21 | 12 | 1 | - | - | - | 167 |
|  | Delirium, n | 3 | 10 | 2 | 3 | 0 | - | - | - | 18 |
| Martinez, 2012 | Risk score (points) | 0 | 1 | 2 | N/A | N/A | N/A | N/A | N/A |  |
|  | N_total_ | 259 | 19 | 4 | - | - | - | - | - | 282 |
|  | Delirium, n | 21 | 2 | 2 | - | - | - | - | - | 25 |
| Kobayashi, 2013 | Risk score | Low | Moderate | High | Quite high | N/A | N/A | N/A | N/A |  |
|  | N_total_ | 125 | 104 | 13 | 41 | - | - | - | - | 283 |
|  | Delirium, n | 7 | 14 | 0 | 4 | - | - | - | - | 25 |

**Table S2.** Number of persons per risk category per risk stratification instrument (continued).

Abbreviations: N/A, not applicable.

^a^ Number <292 due to missing data for some risk factors included by the risk stratification instruments.
