# Supplementary material for: Performance And Agreement Of Risk Stratification Instruments For Postoperative Delirium In Persons Aged 50 Years Or Older
Source: PLoS One. 2014 Dec 2;9(12):e113946. doi: 10.1371/journal.pone.0113946 (PMC4252072; doi:10.1371/journal.pone.0113946)
Supplement: Table S3 — (DOC) [file pone.0113946.s003.doc]

**Table S3.** Positive agreement between the risk stratification instruments for postoperative delirium.^a^

| **Risk stratification instrument (first author, year of publication)** | | **Inouye, 1993** | **Marcantonio, 1994** | **Pompei, 1994** | **O’Keeffe, 1996** | **Freter, 2005** | **Greene, 2009** | **Rudolph, 2009** | **Martinez, 2012** | **Kobayashi, 2013** |
| --- | --- | --- | --- | --- | --- | --- | --- | --- | --- | --- |
| Inouye, 1993 | % agreement | 100 | 20 | 20 | 11 | 14 | 0 | 4 | 0 | 7 |
|  | (95%CI) | (N/A) | (1-39) | (0-53) | (0-21) | (2-27) | (0-0) | (0-9) | (0-0) | (0-16) |
| Marcantonio, 1994 | % agreement | 20 | 100 | 12 | 35 | 28 | 57 | 15 | 12 | 38 |
|  | (95%CI) | (1-39) | (N/A) | (0-27) | (22-49) | (15-41) | (26-88) | (6-24) | (0-25) | (27-48) |
| Pompei, 1994 | % agreement | 20 | 12 | 100 | 3 | 10 | 0 | 4 | 0 | 3 |
|  | (95%CI) | (0-53) | (0-27) | (N/A) | (0-10) | (0-21) | (0-0) | (0-8) | (0-0) | (0-10) |
| O’Keeffe, 1996 | % agreement | 11 | 35 | 10 | 100 | 42 | 40 | 42 | 16 | 36 |
|  | (95%CI) | (0-21) | (22-49) | (0-10) | (N/A) | (30-54) | (13-67) | (31-52) | (5-27) | (24-48) |
| Freter, 2005 | % agreement | 14 | 28 | 10 | 42 | 100 | 20 | 27 | 45 | 50 |
|  | (95%CI) | (2-27) | (15-41) | (0-21) | (30-54) | (N/A) | (0-44) | (17-36) | (31-59) | (39-62) |
| Greene, 2009 | % agreement | 0 | 57 | 0 | 40 | 20 | 100 | 24 | 14 | 9 |
|  | (95%CI) | (0-0) | (26-88) | (0-0) | (13-67) | (0-44) | (N/A) | (5-42) | (0-39) | (0-26) |
| Rudolph, 2009 | % agreement | 4 | 15 | 4 | 42 | 27 | 24 | 100 | 17 | 34 |
|  | (95%CI) | (0-9) | (6-24) | (0-8) | (31-52) | (17-36) | (5-42) | (N/A) | (8-26) | (24-44) |
| Martinez, 2012 | % agreement | 0 | 12 | 0 | 16 | 45 | 14 | 17 | 100 | 29 |
|  | (95%CI) | (0-0) | (0-25) | (0-0) | (5-27) | (31-59) | (0-39) | (8-26) | (N/A) | (15-42) |
| Kobayashi, 2013 | % agreement | 7 | 38 | 3 | 36 | 50 | 9 | 34 | 29 | 100 |
|  | (95%CI) | (0-16) | (27-48) | (0-10) | (24-48) | (39-62) | (0-26) | (24-44) | (15-42) | (N/A) |

Abbreviations: CI, confidence interval; N/A, not applicable.

^a^ Positive agreement is the agreement between two risk stratification instruments in identifying patients at high risk for postoperative delirium. For the cut-off points of the risk stratifiction instruments for low vs. high risk of postoperative delirium, and the calculation of positive agreement and its 95%CI, see text.
