# Supplementary material for: Performance And Agreement Of Risk Stratification Instruments For Postoperative Delirium In Persons Aged 50 Years Or Older
Source: PLoS One. 2014 Dec 2;9(12):e113946. doi: 10.1371/journal.pone.0113946 (PMC4252072; doi:10.1371/journal.pone.0113946)
Supplement: Text S1 — (DOC) [file pone.0113946.s004.doc]

**Text S1. Excluded articles and risk stratification instruments**

**Full text articles excluded**

***Reason: Screening instrument for delirium at day of admission***

- Han JH, Zimmerman EE, Cutler N, Schnelle J, Morandi A, Dittus RS, et al. Delirium in older emergency department patients: recognition, risk factors, and psychomotor subtypes. Acad Emerg Med 2009; 16: 193-200.
- Kennedy M, Enander RA, Tadiri SP, Wolfe RE, Shapiro NI, Marcantonio ER. Delirium risk prediction, healthcare use and mortality of elderly adults in the emergency department. J Am Geriatr Soc 2014; 62: 462-9.
- Ritchie CW, Newman TH, Leurent B, Sampson EL. The association between C-reactive protein and delirium in 710 acute elderly hospital admissions. Int Psychogeriatr 2014; 26: 717-24.

***Reason: Separate risk factors, no risk stratification instrument***

- Afonso A, Scurlock C, Reich D, Raikhelkar J, Hossain S, Bodian C, et al. Predictive model for postoperative delirium in cardiac surgical patients. Semin Cardiothorac Vasc Anesth 2010; 14: 212-7.
- Cheong JA. Diagnosis, risk factors, predisposing factors, and predictive models of delirium. Am J Geriatr Psychiatry 2013; 21: 931-4.
- Wang SH, Wang JY, Lin PY, Lin KH, Ko CJ, Hsieh CE, et al. Predisposing risk factors for delirium in living donor liver transplantation patients in intensive care units. PLoS One 2014; 9: e96676.
- Watanuki S, Takeuchi T, Matsuda Y, Terauchi H, Takahashi Y, Goshima M, et al. Structural visualization of expert nursing: Development of an assessment and intervention algorithm for delirium following abdominal and thoracic surgeries. Stud Health Technol Inform 2006;122: 925-6.

**Risk stratification instruments excluded**

***Reason: Risk factors not identified at hospital admission***

- Bohner H, Hummel TC, Habel U, Miller C, Reinbott S, Yang Q, et al. Predicting delirium after vascular surgery: a model based on pre- and intraoperative data. Ann Surg 2003; 238: 149-56.
- Brouquet A, Cudennec T, Benoist S, Moulias S, Beauchet A, Penna C, et al. Impaired mobility, ASA status and administration of tramadol are risk factors for postoperative delirium in patients aged 75 years or more after major abdominal surgery. Ann Surg 2010; 251: 759-65.
- Dai YT, Lou MF, Yip PK, Huang GS. Risk factors and incidence of postoperative delirium in elderly Chinese patients. Gerontology 2000; 46: 28-35.
- Guenther U, Theuerkauf N, Frommann I, Brimmers K, Malik R, Stori S, et al. Predisposing and precipitating factors of delirium after cardiac surgery: a prospective observational cohort study. Ann Surg 2013; 257:1160-7.
- Inouye SK. Predisposing and precipitating factors for delirium in hospitalized older patients. Dement Geriatr Cogn Disord 1999; 10: 393-400.
- Inouye SK, Charpentier PA. Precipitating factors for delirium in hospitalized elderly persons. Predictive model and interrelationship with baseline vulnerability. JAMA 1996; 275: 852-7.
- Katznelson R, Djaiani GN, Borger MA, Friedman Z, Abbey SE, Fedorko L, et al. Preoperative use of statins is associated with reduced early delirium rates after cardiac surgery. Anesthesiology 2009; 110: 67-73.
- Kostalova M, Bednarik J, Mitasova A, Dusek L, Michalcakova R, Kerkovsky M, et al. Towards a predictive model for post-stroke delirium. Brain Inj 2012; 26: 962-71.
- Krzych LJ, Wybraniec MT, Krupka-Matuszczyk I, Skrzypek M, Bochenek AA. Delirium Screening in Cardiac Surgery (DESCARD): A Useful Tool for Nonpsychiatrists. Can J Cardiol 2014; 30: 932‑9. (as for perioperative risk stratification instrument)
- Krzych LJ, Wybraniec MT, Krupka-Matuszczyk I, Skrzypek M, Bolkowska A, Wilczynski M, et al. Complex assessment of the incidence and risk factors of delirium in a large cohort of cardiac surgery patients: a single-center 6-year experience. Biomed Res Int 2013; 2013: 835850.
- Levkoff SE, Safran C, Cleary PD, Gallop J, Phillips RS. Identification of factors associated with the diagnosis of delirium in elderly hospitalized patients. J Am Geriatr Soc 1988; 36: 1099-104.
- Shah S, Weed HG, He X, Agrawal A, Ozer E, Schuller DE. Alcohol-related predictors of delirium after major head and neck cancer surgery. Arch Otolaryngol Head Neck Surg 2012; 138: 266-71.
- Smulter N, Lingehall HC, Gustafson Y, Olofsson B, Engstrom KG. Delirium after cardiac surgery: incidence and risk factors. Interact Cardiovasc Thorac Surg 2013; 17: 790-6.
- Wragg RE, Dimsdale JE, Moser KM, Daily PO, Dembitsky WP, Archibald C. Operative predictors of delirium after pulmonary thromboendarterectomy. A model for postcardiotomy delirium?. J Thorac Cardiovasc Surg 1988; 96: 524-9.

***Reason: Risk stratification instrument not validated in independent cohort***

- Alagiakrishnan K, Marrie T, Rolfson D, Coke W, Camicioli R, Duggan D, et al. Simple cognitive testing (Mini-Cog) predicts in-hospital delirium in the elderly. J Am Geriatr Soc 2007; 55: 314-6.
- Fisher BW, Flowerdew G. A simple model for predicting postoperative delirium in older patients undergoing elective orthopedic surgery. J Am Geriatr Soc 1995; 43: 175-8.
- Francis J, Martin D, Kapoor WN. A prospective study of delirium in hospitalized elderly. JAMA 1990; 263: 1097-101.
- Franco JG, Valencia C, Bernal C, Ocampo MV, Trzepacz PT, Pablo J, et al. Relationship between cognitive status at admission and incident delirium in older medical inpatients. J Neuropsychiatry Clin Neurosci 2010; 22: 329-37.
- Freter SH, George J, Dunbar MJ, Morrison M, Macknight C, Rockwood K. Prediction of delirium in fractured neck of femur as part of routine preoperative nursing care. Age Ageing 2005; 34:387-8.
- Goldenberg G, Kiselev P, Bharathan T, Baccash E, Gill L, Madhav V, et al. Predicting post-operative delirium in elderly patients undergoing surgery for hip fracture. Psychogeriatrics 2006; 6: 43-8.
- Harasawa N, Mizuno T. A novel scale predicting postoperative delirium (POD) in patients undergoing cerebrovascular surgery. Arch Gerontol Geriatr 2014; 59: 264-71.
- Hattori H, Kamiya J, Shimada H, Akiyama H, Yasui A, Kuroiwa K, et al. Assessment of the risk of postoperative delirium in elderly patients using E-PASS and the NEECHAM Confusion Scale. Int J Geriatr Psychiatry 2009; 24: 1304-10.
- Isfandiaty R, Harimurti K, Setiati S, Roosheroe AG. Incidence and predictors for delirium in hospitalized elderly patients: a retrospective cohort study. Acta Med Indones 2012; 44: 290-7.
- Koster S, Hensens AG, Schuurmans MJ, van der Palen J. Prediction of delirium after cardiac surgery and the use of a risk checklist. Eur J Cardiovasc Nurs 2013; 12: 284-92.
- Leung JM, Tsai TL, Sands LP. Brief report: preoperative frailty in older surgical patients is associated with early postoperative delirium. Anesth Analg 2011; 112: 1199-201.
- Litaker D, Locala J, Franco K, Bronson DL, Tannous Z. Preoperative risk factors for postoperative delirium. Gen Hosp Psychiatry 2001; 23: 84-9.
- Macdonald A, Adamis D, Treloar A, Martin F. C-reactive protein levels predict the incidence of delirium and recovery from it. Age Ageing 2007; 36: 222-5.
- McGuire JM. The incidence of and risk factors for emergence delirium in U.S. military combat veterans. J Perianesth Nurs 2012; 27: 236-45.
- Meziere A, Paillaud E, Belmin J, Pariel S, Herbaud S, Canoui-Poitrine F, et al. Delirium in older people after proximal femoral fracture repair: role of a preoperative screening cognitive test. Ann Fr Anesth Reanim 2013; 32: e91-6.
- Moerman S, Tuinebreijer WE, de Boo M, Pilot P, Nelissen RG, Vochteloo AJ. Validation of the Risk Model for Delirium in hip fracture patients. Gen Hosp Psychiatry 2012; 34: 153-9.
- Pol RA, van Leeuwen BL, Visser L, Izaks GJ, van den Dungen JJ, Tielliu IF, et al. Standardised frailty indicator as predictor for postoperative delirium after vascular surgery: a prospective cohort study. Eur J Vasc Endovasc Surg 2011; 42: 824-30.
- Priner M, Jourdain M, Bouche G, Merlet-Chicoine I, Chaumier JA, Paccalin M. Usefulness of the short IQCODE for predicting postoperative delirium in elderly patients undergoing hip and knee replacement surgery. Gerontology 2008; 54: 116-9.
- Rudolph JL, Jones RN, Rasmussen LS, Silverstein JH, Inouye SK, Marcantonio ER. Independent vascular and cognitive risk factors for postoperative delirium. Am J Med 2007; 120: 807-13.
- Zhang Z, Pan L, Deng H, Ni H, Xu X. Prediction of delirium in critically ill patients with elevated C-reactive protein. J Crit Care 2014; 29: 88-92.

***Reason: Intensive Care/ Stroke Care Unit setting***

- Oldenbeuving AW, de Kort PL, van Eck van der Sluijs,J.F., Kappelle LJ, Roks G. An early prediction of delirium in the acute phase after stroke. J Neurol Neurosurg Psychiatry 2014; 85: 431-4.
- van den Boogaard M, Pickkers P, Slooter AJ, Kuiper MA, Spronk PE, van der Voort PH, et al. Development and validation of PRE-DELIRIC (PREdiction of DELIRium in ICu patients) delirium prediction model for intensive care patients: observational multicentre study. BMJ 2012; 344: e420.

***(Alternative) data on risk factors not available***

- Carrasco MP, Villarroel L, Andrade M, Calderon J, Gonzalez M. Development and validation of a delirium predictive score in older people. Age Ageing 2014; 43: 346-51.
- Douglas VC, Hessler CS, Dhaliwal G, Betjemann JP, Fukuda KA, Alameddine LR, et al. The AWOL tool: derivation and validation of a delirium prediction rule. J Hosp Med 2013; 8: 493-9.
- Koster S, Oosterveld FG, Hensens AG, Wijma A, van der Palen J. Delirium after cardiac surgery and predictive validity of a risk checklist. Ann Thorac Surg 2008; 86: 1883-7.
- Krzych LJ, Wybraniec MT, Krupka-Matuszczyk I, Skrzypek M, Bochenek AA. Delirium Screening in Cardiac Surgery (DESCARD): A Useful Tool for Nonpsychiatrists. Can J Cardiol 2014; 30: 932‑9. (as for preoperative risk stratification instrument)
- Leung JM, Sands LP, Lim E, Tsai TL, Kinjo S. Does preoperative risk for delirium moderate the effects of postoperative pain and opiate use on postoperative delirium?. Am J Geriatr Psychiatry 2013; 21: 946-56.
