# Supplementary material for: Performance And Agreement Of Risk Stratification Instruments For Postoperative Delirium In Persons Aged 50 Years Or Older
Source: PLoS One. 2014 Dec 2;9(12):e113946. doi: 10.1371/journal.pone.0113946 (PMC4252072; doi:10.1371/journal.pone.0113946)
Supplement: Text S2 — (DOC) [file pone.0113946.s005.doc]

**Text S2: Sensitivity analyses**

We performed several sensitivity analyses. First, the analysis was repeated in a subsample that only included the persons aged 60 years or older (Table A). In addition, the analysis was repeated (in the total study population) using different definitions of comorbidity (Table B), dependency in activities of daily living (ADL)(Table C), and impairment in executive function (Table D).

**Table A.** Performance of the risk stratification instruments in identifying patients at high risk for postoperative delirium: analysis limited to persons aged ≥60 years.^a, b^

| **Risk stratification instrument (first author, year of publication)** | **Sensitivity (%)** | **Specificity (%)** | **Positive predictive value (%)** | **Negative predictive value (%)** | **ROC analysis** | |
| --- | --- | --- | --- | --- | --- | --- |
|  |  |  |  |  | **AUC (95%CI)** | ***p*-value** |
| Inouye, 1993 | 0 | 98 | 0 | 90 | 0.47 (0.32-0.62) | 0.64 |
| Marcantonio, 1994 | 20 | 89 | 17 | 91 | 0.52 (0.37-0.66) | 0.78 |
| Pompei, 1994 | 0 | 97 | 0 | 90 | 0.51 (0.39-0.63) | 0.86 |
| O'Keeffe, 1996 | 25 | 80 | 13 | 90 | 0.52 (0.39-0.66) | 0.73 |
| Freter, 2005 | 24 | 79 | 11 | 90 | 0.56 (0.43-0.69) | 0.39 |
| Greene, 2009 | 20 | 89 | 25 | 86 | 0.63 (0.45-0.81) | 0.18 |
| Rudolph, 2009 | 79 | 31 | 13 | 92 | 0.57 (0.41-0.73) | 0.39 |
| Martinez, 2012 | 14 | 91 | 16 | 90 | 0.53 (0.40-0.67) | 0.63 |
| Kobayashi, 2013 | 19 | 79 | 9 | 90 | 0.54 (0.42-0.67) | 0.52 |

Abbreviations: AUC: area under the curve; CI: confidence interval; ROC: receiver operating characteristic.

^a^ For the cut-off points of the risk stratification instruments for low vs. high risk, see text.

^b^ The incidence of delirium in persons aged ≥60 years was 10% (95%CI, 7%-15%).

^c^ the mean age (SD) of this subsample was 69 (7) years.

**Table B.** Performance of the risk stratification instruments in identifying patients at high risk for postoperative delirium: different definitions of comorbidity.^a^

| **Risk stratification instrument (first author, year of publication)** | **Sensitivity (%)** | **Specificity (%)** | **Positive predictive value (%)** | **Negative predictive value (%)** | **ROC analysis** | |
| --- | --- | --- | --- | --- | --- | --- |
| Definition of comorbidity^b^ |  |  |  |  | **AUC (95%CI)** | ***p*-value** |
| **Pompei, 1994** |  |  |  |  |  |  |
| ≥2 diseases | 0 | 98 | 0 | 91 | 0.54 (0.43-0.65) | 0.49 |
| ≥3 diseases | 0 | 98 | 0 | 91 | 0.53 (0.42-0.64) | 0.58 |
| ≥4 diseases | 0 | 100 | 0 | 91 | 0.50 (0.38-0.62) | 0.99 |

Abbreviations: AUC: area under the curve; CI: confidence interval; ROC: receiver operating characteristic.

^a^ For the cut-off points of the risk stratification instruments for low vs. high risk, see text.

^b^ Comorbidity was only included in the risk stratification instrument of Pompei, 1994 [1].

**Table C.** Performance of the risk stratification instruments in identifying patients at high risk for postoperative delirium: different definitions of dependency in ADL.^a^

| **Risk stratification instrument (first author, year of publication)** | **Sensitivity (%)** | **Specificity (%)** | **Positive predictive value (%)** | **Negative predictive value (%)** | **ROC analysis** | |
| --- | --- | --- | --- | --- | --- | --- |
| Definition of dependency in ADL^b^ |  |  |  |  | **AUC (95%CI)** | ***p*-value** |
| **Freter, 2005** |  |  |  |  |  |  |
| Dependent in ≥1 out of 4 activities | 36 | 80 | 15 | 93 | 0.58 (0.46-0.71) | 0.18 |
| Dependent in ≥1 out of 3 activities | 28 | 82 | 13 | 92 | 0.55 (0.43-0.67) | 0.40 |
| Dependent in ≥2 out of 3 activities | 24 | 84 | 13 | 92 | 0.54 (0.42-0.66) | 0.53 |
| **Martinez, 2012** |  |  |  |  |  |  |
| Dependent in ≥1 out of 3 activities | 24 | 88 | 17 | 92 | 0.56 (0.44-0.69) | 0.31 |
| Dependent in ≥2 out of 3 activities | 16 | 93 | 17 | 92 | 0.54 (0.42-0.67) | 0.48 |
| Dependent in ≥3 out of 3 activities | 16 | 94 | 20 | 92 | 0.55 (0.42-0.67) | 0.42 |
| **Kobayashi, 2013** |  |  |  |  |  |  |
| Dependent in ≥1 out of 5 activities | 16 | 81 | 7 | 91 | 0.57 (0.46-0.67) | 0.27 |
| Dependent in ≥1 out of 5 activities | 16 | 83 | 9 | 91 | 0.59 (0.48-0.69) | 0.16 |
| Dependent in ≥1 out of 5 activities | 16 | 84 | 9 | 91 | 0.59 (0.48-0.70) | 0.13 |

Abbreviations: ADL: activities of daily life; AUC: area under the curve; CI: confidence interval; ROC: receiver operating characteristic.

^a^ For the cut-off points of the risk stratification instruments for low vs. high risk, see text.

^b^ Dependency in activities of daily life (ADL) was included in the risk stratification instruments of Freter, 2005 [2,3], Martinez, 2012 [4], and Kobayashi, 2013 [5].

**Table D.** Performance of the risk stratification instruments in identifying patients at high risk for postoperative delirium: different definitions of impairment in executive function.^a^

| **Risk stratification instrument (first author, year of publication)** | **Sensitivity (%)** | **Specificity (%)** | **Positive predictive value (%)** | **Negative predictive value (%)** | **ROC analysis** | |
| --- | --- | --- | --- | --- | --- | --- |
| Definition of impairment in executive function^b^ |  |  |  |  | **AUC (95%CI)** | ***p*-value** |
| **Greene, 2005** |  |  |  |  |  |  |
| Letterfluency ≤3 words/min | 10 | 96 | 25 | 88 | 0.66 (0.49-0.84) | 0.09 |
| Letterfluency ≤5 words/min | 20 | 92 | 25 | 89 | 0.66 (0.48-0.83) | 0.11 |
| Letterfluency ≤7 words/min | 20 | 87 | 18 | 89 | 0.67 (0.52-0.81) | 0.09 |

Abbreviations: AUC: area under the curve; CI: confidence interval; min: minute; ROC: receiver operating characteristic.

^a^ For the cut-off points of the risk stratification instruments for low vs. high risk, see text.

^b^ Impairment of executive function was only included in the risk stratification instrument of Greene, 2009 [6].

**References**

1. Pompei P, Foreman M, Rudberg MA, Inouye SK, Braund V, et al. (1994) Delirium in hospitalized older persons: outcomes and predictors. J Am Geriatr Soc 42: 809-815.
2. Freter SH, Dunbar MJ, MacLeod H, Morrison M, MacKnight C, et al. (2005) Predicting post-operative delirium in elective orthopaedic patients: the Delirium Elderly At-Risk (DEAR) instrument. Age Ageing 34: 169-171.
3. Freter SH, George J, Dunbar MJ, Morrison M, Macknight C, et al. (2005) Prediction of delirium in fractured neck of femur as part of routine preoperative nursing care. Age Ageing 34: 387-388.
4. Martinez JA, Belastegui A, Basabe I, Goicoechea X, Aguirre C, et al. (2012) Derivation and validation of a clinical prediction rule for delirium in patients admitted to a medical ward: an observational study. BMJ Open 2: 10.1136/bmjopen-2012-001599.
5. Kobayashi D, Takahashi O, Arioka H, Koga S, Fukui T (2013) A prediction rule for the development of delirium among patients in medical wards: Chi-Square Automatic Interaction Detector (CHAID) decision tree analysis model. Am J Geriatr Psychiatry 21: 957-962.
6. Greene NH, Attix DK, Weldon BC, Smith PJ, McDonagh DL, et al. (2009) Measures of executive function and depression identify patients at risk for postoperative delirium. Anesthesiology 110: 788-795.
